# Role of Composition and Temperature in Shaping the Structural and Optical Properties of Iodide-Based Hybrid Perovskite Thin Films Produced by PVco-D Technique

**DOI:** 10.3390/ma18061336

**Published:** 2025-03-18

**Authors:** Agnieszka Marjanowska, Krzysztof Wiśniewski, Przemysław Płóciennik, Bouchta Sahraoui, Anna Zawadzka

**Affiliations:** 1 Institute of Physics, Faculty of Physics, Astronomy and Informatics, Nicolaus Copernicus University in Torun, Grudziadzka 5, 87-100 Torun, Poland; wisniek@fizyka.umk.pl; 2 Centre for Modern Interdisciplinary Technologies, Nicolaus Copernicus University, Wilenska 4, 87-100 Torun, Poland; przemas@fizyka.umk.pl; 3 LPhiA, SFR MATRIX, University of Angers, Bd Lavoisier 2, CEDEX 2, 49045 Angers, France; bouchta.sahraoui@univ-angers.fr; 4 Institute of Engineering and Technology, Faculty of Physics, Astronomy, and Informatics, Nicolaus Copernicus University in Torun, Wilenska 7, 87-100 Torun, Poland

**Keywords:** iodide-based hybrid perovskites, PVD, thin films, surface topography, phase transition

## Abstract

The research considered in this publication aims to contribute to developing perovskite-based technologies by conducting basic research on perovskite materials. The materials described in the paper are thin films of hybrid perovskite MEAPbI_3_ made using the PVco-D method in three different compositions—perovskite samples differ in the percentage of organic methylammonium and inorganic iodide parts. This publication discusses the influence of the composition of the thin perovskite layer on its structural and optical properties and the influence of the temperature of the environment of the perovskite thin film on optical properties. To answer these questions, the surface topography was analyzed using the AFM method, spectroscopic measurements were carried out in the UV-Vis-NIR range, and photoluminescence measurements were performed in a wide temperature range—from about 80 K to 310 K. The results indicate that the composition changes the surface topography, forming increasingly higher crystallites (up to 606%) with increasing methylammonium content. The transition temperature from the orthorhombic to the tetragonal phase was determined for each composition at about 140 K. For the composition of 30% MEAI + 70% PbI2, the phase transition temperature from tetragonal to cubic was determined at a temperature close to RT.

## 1. Introduction

Rose, in his work from the first half of the 19th century, was the first to describe perovskites [1]. They began to attract the attention of researchers only in the 1950s. Over time, laboratories developed a method for the chemical synthesis of perovskites [2], but only in the 1970s did Weber from the University of Stuttgart discover the first hybrid, organic–inorganic halide perovskite [3]. The most groundbreaking moment was Japanese researchers’ use of a thin perovskite layer as an active layer in the structure of a photovoltaic cell in 2009. The photovoltaic cell that Kojima et al. were studying at the time achieved an efficiency of 3.8% [4]. Recently, Liu et al. presented results confirming that their cell can achieve an efficiency of 33.9% [5]. Apart from their vast potential in photovoltaic applications, it was proven several years ago by, among others, Mirershadi et al., Marjanowska et al., and Waszkowska et al., that perovskites can be used in nonlinear optics [6,7,8].

Intensive research is still being conducted on hybrid organic–inorganic halide perovskites [9,10,11]. Research and applications are mainly focused on photovoltaic applications, as exemplified by scientists such as Zawadzka et al., Han et al., and Tsvetkov et al. [12,13,14]. Still, there is also no shortage of works describing the various applications of perovskites. Wei et al. and Zhang et al. studied the application of perovskites in the area of lasers [15,16], Fakharuddin et al. in the area of light-emitting diodes [17], Wang et al. in the area of photodetectors [18], Li et al. and Hua et al. in the area of optical switches [19,20] and Hu et al. in the area of high-resolution imaging [21]. The number of scientific publications on perovskite materials and their applications is growing yearly, but a more detailed analysis of their fundamental photophysical properties is still needed.

The most popular hybrid halide perovskite is methylammonium lead iodide (CH_3_NH_3_PbI_3_ or MEAPbI_3_). Due to its unique optoelectronic properties, such as a long carrier lifetime, high carrier mobility, low carrier recombination coefficient, and high absorption coefficient in the UV-Vis range, its applications are often described in the literature [22,23,24]. Despite the large number of published scientific papers, the fundamental photophysical properties of MEAPbI_3_ are not sufficiently well understood and explained. This results in the slow implementation of innovative technical solutions based on perovskites, such as perovskite photovoltaic cells. Therefore, this work addresses the detailed study of the structural and photo-optical properties of thin films of the hybrid organic–inorganic halide perovskite MEAPbI_3_ with different compositions. The UV-Vis-NIR transmission spectra, surface topography performed using the atomic force microscopy technique, and photoluminescence spectra were investigated and described. All tested samples were prepared using the innovative PVco-D technique (physical vapor deposition in co-deposition mode).

The spin-coating technique is the basic technique for producing thin film structures. It consists of dripping a solution of the applied material onto a previously cleaned substrate and causing the substrate with the solution to rotate rapidly around a vertical axis. As a result of rotation, the solution is distributed on the substrate surface and evaporates, leaving a thin layer of material on it. Applying a layer using the spin-coating method takes a relatively short time, and its undoubted advantage is its low level of complexity. The disadvantages of this method of obtaining thin films include the lack of possibility to control the structural properties and thickness of the layer during its formation and the lack of repeatability of the obtained structures. The method of obtaining low-dimensional structures proposed in this article was described by Zawadzka et al. [12]. It is the innovative PVco-D method, which aims to improve the quality of the obtained thin layers by allowing for the simultaneous application of several materials, control of the sublimation rate of the applied materials, and control of the pressure and temperature conditions inside the vacuum chamber during the application process and by obtaining uniform layers with repeatable physicochemical properties.

The presented work aims to investigate and describe the basic structural and photo-optical properties of thin films of the hybrid organic–inorganic halide perovskite MEAPbI_3_. All of the low-dimensional structures were made using the PVco-D technique. This innovative method allowed the production of samples with different compositions. The obtained research results allow for the exploration of information on the fundamental properties of the MEAPbI_3_ perovskite, which have not been sufficiently understood so far, for example, changes in the transmission and photoluminescence spectra in a wide temperature range of 80 K–310 K. In addition, studies of the surface topography of thin films provide information on their nature, how they are formed, and their quality. A comparison of the results corresponding to different compositions of perovskite structures allows for the determination of their optimal composition to obtain the best photo-optical and optoelectronic parameters. Looking at the presented research results more broadly, knowledge of the fundamental properties of the MEAPbI_3_ perovskite will facilitate the design of optoelectronic devices based on such materials. The research results will contribute to the development of perovskite technologies in industry, especially in the area of perovskite photovoltaic cells, and also, due to the use of low-temperature and vacuum conditions in research, will pave the way to applications in space.

## 2. Materials and Methods

### 2.1. Materials

Hybrid organic–inorganic halide perovskites are described by the general formula ABX_3_. At the A site, there is a cation, which is the non-binding monovalent organic methylammonium cation CH_3_NH_3_^+^ for the perovskites studied in this work. The B site is occupied by an octahedral coordinated divalent metal ion Pb^2+^, and a monoammonium halide ion I occupies the X site. Perovskites are a group of minerals that can be distinguished from many others by a specific crystal lattice defining their physicochemical properties. Moreover, they can change in the crystal lattice due to temperature. Such changes in the crystal structure affect the physicochemical properties of the material and are called phase transitions.

**Figure 1:**
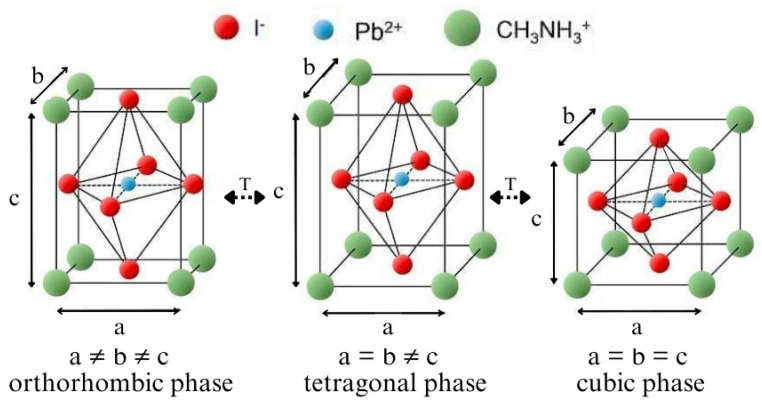
The crystal structures of MEAPbI_3_: orthorhombic phase, tetragonal phase, and cubic phase.

Zawadzka et al., Li et al., and Zhang et al. in their works claim that perovskite materials can occur in three crystallographic phases: cubic, tetragonal, and orthorhombic [12,25,26] (Figure 1). The ability to change the phase is possible by changing the temperature of the material, and each phase change affects its optical parameters. According to researchers, perovskite assumes the tetragonal phase at room temperature. At temperatures higher than room temperature, it occurs in the cubic and orthorhombic phases at lower temperatures. Phase transition temperatures are conventional for the group of perovskite materials. There are various types of perovskites that can be obtained by chemical synthesis, which allows for tuning their physicochemical properties. Therefore, the temperatures corresponding to phase transitions can also be different. According to sources provided by Brittman et al., MEAPbI_3_ assumes the tetragonal phase at room temperature, the transition to the cubic phase is observed at 330–350 K, and the transition to the orthorhombic phase is observed at about 140–160 K [27].

**Figure 2:**
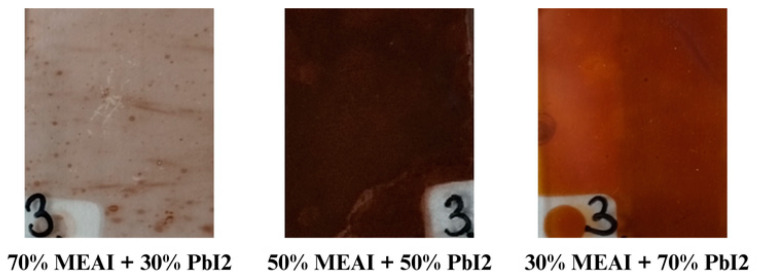
Photographs of tested samples measuring approximately 2 cm × 2.5 cm.

The tested samples were thin films of MEAPbI_3_ hybrid perovskites. The samples were made using the PVco-D method by the co-deposition of methylammonium iodide and lead (II) iodide on a glass substrate. They differed in the percentage composition of both parts and the thickness of the film. Three compositions were selected for the tests—30% MEAI + 70% PbI_2_, 70% MEAI + 30% PbI_2_, and 50% MEAI + 50% PbI_2_, with thicknesses of 50 nm, 180 nm, and 285 nm, respectively (Figure 2).

### 2.2. Methods

Thin film samples of the studied perovskite material were made using the physical vapor deposition technique in the co-deposition mode. The studies used measurement methods such as atomic force microscopy, UV-Vis-NIR spectroscopy, and photoluminescence spectroscopy. Spectroscopic measurements were performed in a wide temperature range from about 80 K to 310 K, which was possible thanks to the use of a cryostat with liquid nitrogen and a temperature controller.

#### 2.2.1. Physical Vapor Deposition

Physical vapor deposition (PVD) is a technique of depositing 2D low-dimensional structures in high-vacuum conditions on a previously prepared substrate. The possibility of carrying out the PVD process in the co-deposition mode (PVco-D) is an original idea of our research group. We aimed to create a high-quality thin film structure in controlled deposition conditions characterized by physicochemical properties other than those of the component materials [7]. The co-deposition process makes it possible to create various new materials, including hybrid perovskites.

**Figure 3:**
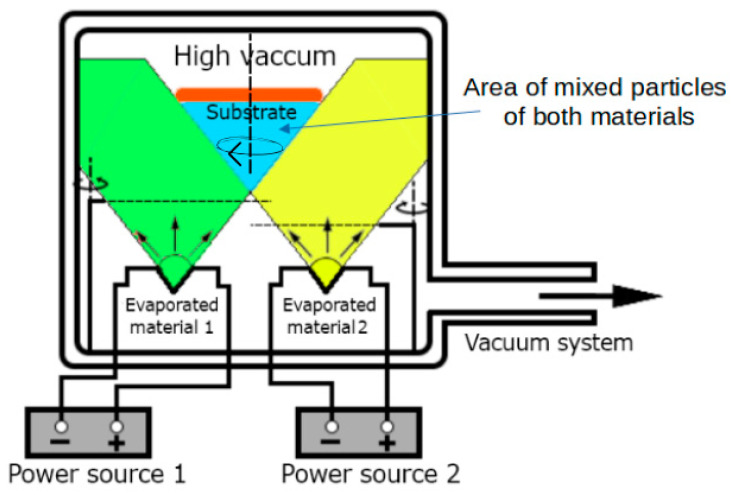
Schematic presentation of the PVco-D process [7].

The thin layers described in the paper were created using the Thin Film Deposition System—NANO 36™ (Kurt J. Lesker Company, Jefferson Hills, PA, USA), equipped with two independently working thermal sources. The apparatus diagram is shown in Figure 3. This work used quartz glass substrates (BK7) with surfaces of about 4 cm^2^. The entire process of thin layer deposition takes place in a tightly closed vacuum chamber under high-vacuum conditions of the order of 10^−5^ Tr. To obtain the tested thin films of hybrid perovskites, we used methylammonium iodide (organic part) and lead (II) iodide (inorganic part), the melting points of which are different. It is about 280 °C for methylammonium iodide, and, for lead (II) iodide, it is about 400 °C. The shutters and piezoelectric thickness sensors placed inside the chamber and the possibility of controlling the conditions of pressure, temperature, and evaporation rate of materials allow for the precise control of the deposition process and the creation of the desired thin film structure of a specific thickness and composition.

The PVco-D technique is a handy tool compared to other thin film techniques. Firstly, it allows the obtention of perovskite hybrid structures with more than one component with different sublimation temperatures, and it is impossible to obtain these structures using other known deposition techniques. The second huge advantage is the ability to control the deposition conditions, thanks to which the resulting structures have precisely the composition that we desire. The possibility of control also affects the repeatability of the obtained layers. In addition, high-vacuum conditions during the entire deposition process guarantee the purity of the obtained structures and, therefore, their high quality and uniformity. High-vacuum conditions also provide appropriate conditions for sublimation and thermally isolate the vapor sources.

#### 2.2.2. Atomic Force Microscopy

To determine the surface roughness and structural properties of the studied MEAPbI_3_ hybrid perovskite thin films, atomic force microscopy (AFM) and Minkowski functional analysis were performed. AFM imaging was performed in contact mode using the NanoSurf EasyScan 2 instrument with a Sicon-A cantilever (Zollikofen, Switzerland).

The AFM measurement technique is a microscopic technique for imaging surface topography and is used in research by, among others, Anoua et al. and Krbaťa et al. [28,29]. AFM allows us to learn about the surface topography of the tested sample, its structure, its roughness, the crystallite sizes, or the type of tested surface. Korpi et al. used the Minkowski functional method (MFM) for more detailed analysis [30]. It is used, among others, to determine the spatial distribution of holes or crystallites on the tested surface or how the structure grows. Among the MFs, there are three distinguished, Minkowski Volume V(h), Minkowski Boundary S(h), and Minkowski Connectivity Χ(h), which are defined as follows:

(1) $$V\left(h\right)=\frac{N_{B}}{N_{B}{+N}_{W}},$$

(2) $$S\left(h\right)=\frac{N_{\mathrm{Bounded}}}{N_{B}{+N}_{W}},$$

(3) $$Χ\left(h\right)=\frac{n_{B}-n_{W}}{N_{B}{+N}_{W}}$$

where

h—height relative to the ground at which the topography of the layer is analyzed;
N_B_—material points;
N_W_—empty points (air);
N_Bounded_—limited pixels;
n_B_—number of isolated areas at height h;
n_W_—number of isolated islands at height h.

#### 2.2.3. Spectroscopic Measurements

To characterize the tested materials, spectroscopic measurements were performed—transmission and photoluminescence spectrum measurements. Both measurements were performed in a wide temperature range from about 80 K to 310 K with a step of 10 K to observe changes occurring in thin perovskite layers. According to theoretical predictions, perovskite structures are characterized by phase transitions at specific temperatures. These changes are reflected in the recorded spectra. By collecting spectra for different temperatures, differences between them are observed, which indicate phase transitions occurring in the tested low-dimensional structures. Knowledge of the optical properties of thin layers, such as, among others, the optical energy gap *Eg*, the reflection coefficient *n*, the absorption coefficient *a*, or phase transitions, is essential, especially from the point of view of optical and optoelectronic applications.

Transmission spectra of thin perovskite films were recorded for wavelengths ranging from 250 nm to 1100 nm with a resolution of 1 nm using an Analytik Jena UV-Vis-NIR spectrometer (Jena, Germany). To investigate changes conditioned by the sample’s ambient temperature, a Janis SuperTran-VP cryostat (Wilmington, MA, USA), a Lake Shore Cryotronics temperature controller (Westerville, OH, USA), and a vacuum pump with a pressure sensor were connected to the measuring system. Liquid nitrogen was the cooling medium, and thanks to the vacuum system, all measurements were performed under a pressure of the order of 10^−3^ Tr. Due to the temperature of liquid nitrogen (~77 K) and hardware limitations, the measurements were performed in the temperature range of 80–310 K, with a step of 10 K, starting from the lowest temperature.

**Figure 4:**
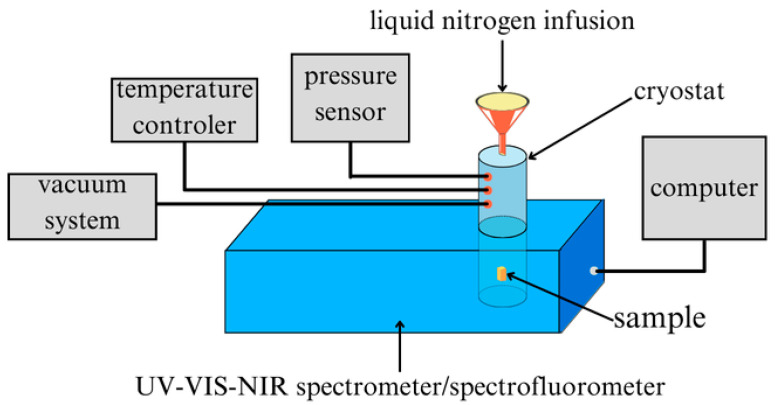
Schematic diagram of the apparatus for UV-Vis-NIR spectroscopic measurements and photoluminescence as a function of temperature.

For the photoluminescence (PL) spectrum measurements, the measuring device was the FluoroMax-4P HORIBA Spectrofluorometer (Kyoto, Japan), working in a measuring system together with a Janis SuperTran-VP cryostat, a Lake Shore Cryotronics temperature controller (Westerville, OH, USA), and a vacuum pump with a pressure sensor (Figure 4). PL spectra were recorded for wavelengths of 550–860 nm, and, as in the case of UV-Vis-NIR measurements, as a function of temperature in the 80–310 K temperature range, starting from the lowest temperature (measurements with a 10 K step). The cooling medium in the cryostat was liquid nitrogen, and measurements were made under a pressure of 10^−3^ Tr.

## 3. Results and Discussion

### 3.1. Surface Topography Characterization

**Figure 5:**
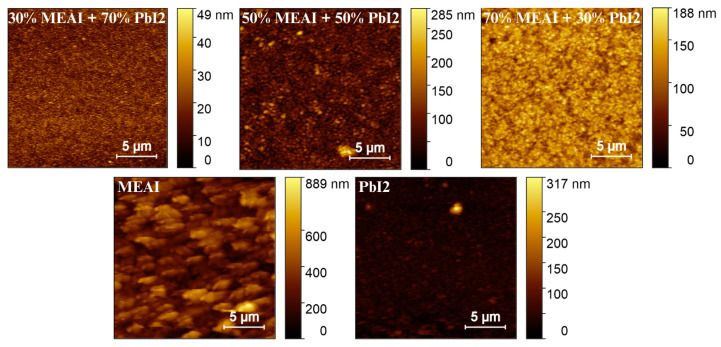
AFM images of MEAPbI_3_ perovskite thin films of three different compositions and MEAI and PbI_2_ thin films.

**Figure 6:**
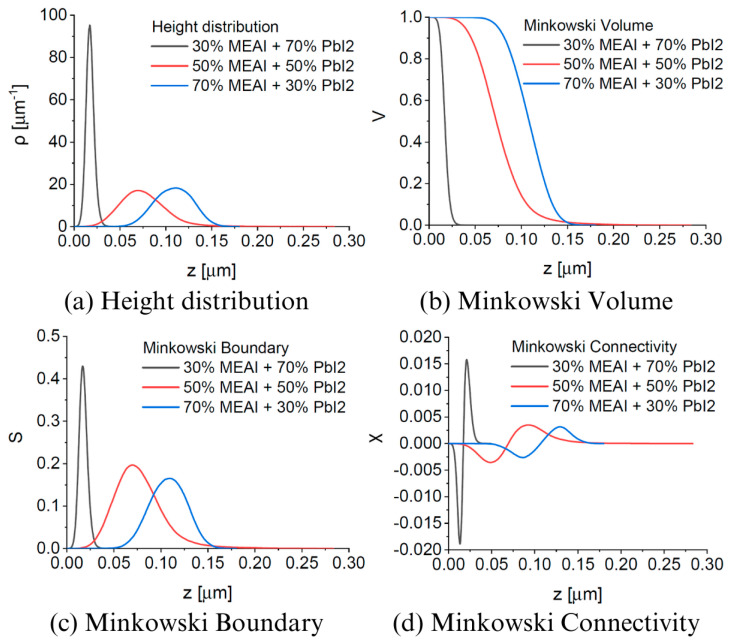
Parameters of MEAPbI_3_ perovskite thin films for different compositions determined based on AFM measurements: (**a**) height distribution; (**b**) Minkowski Volume; (**c**) Minkowski Boundary; (**d**) Minkowski Connectivity.

AFM measurements were performed to investigate thin film samples’ surface topography and microstructure. The obtained results allowed for the generation of 3D images reflecting the topography of the studied surfaces, the determination of parameters characterizing the layers such as “Average crystallite height”, “Mean square roughness”, and “Height distribution”, and analysis using the Minkowski functional method (MFM). The AFM data analysis was performed using the Gwyddion 2.67 computer software, and the results are presented in Figure 5 and Figure 6 and Table 1. Two 3D AFM images are included in Figure 5, and information on the pure MEAI and PbI_2_ thin films is added in Table 1 to compare these layers with the studied perovskite thin films. The analyzed surfaces had dimensions of 20 µm × 20 µm.

AFM images of thin perovskite films show that the tested samples are microcrystalline, and the sizes of single crystallites increase from several dozen nanometers to even 1000 nm with the increase in MEAI content in the perovskite structure. Increasing the content of the methylammonium part and reducing the content of PbI_2_ also increases the average crystallite height and roughness of the thin film. In the case of increasing the MEAI content in the tested perovskite from 30% to 70%, the roughness of the thin film increases by more than 60%. The 30% MEAI + 70% PbI_2_ film is characterized by many single, small crystallites resembling evenly distributed pins. A few single crystallites, higher than the other crystallites, appear on its surface. The structure with the composition of 50% MEAI + 50% PbI_2_ is composed of larger crystallites, and more individual, higher structures appear on its surface than the other ones, which contributes to the increase in roughness. The third composition of 70% MEAI + 50% PbI_2_ is characterized by larger, evenly distributed crystallites on the surface, with very similar heights. Hidalgo et al., in their article, determine that the roughness (RMS) of a thin MAPbI_3_ perovskite layer formed from a solution oscillates from 10 nm to 20 nm depending on the solvent used [31]. Their range of RMS values is very close to those determined in this work. It is worth remembering that the methods of obtaining thin layers in this work and in the work of Hidalgo et al. differ, and this may be the main factor introducing differences in the results obtained. In MFM analysis, V(h), S(h), and Χ(h) provide additional information on the tested microstructures. Based on the shape of V(h), it is concluded that the percolation threshold increases from about 0.01 µm to 0.07 µm with increasing MEAI content, which means that the tested sample has a compact structure from the substrate side to this determined height level (percolation threshold). The shape of the Χ(h) characteristic, otherwise called the Euler characteristic, provides information on the nature of the thin layer formed. The positive part of Χ(h) indicates the island nature of the layer, and the negative part indicates the porous nature. The maximum of the characteristic and its minimum provide information on the maximum density of peaks and the maximum density of valleys, respectively. The positive and negative parts of Χ(h) have a more symmetrical shape concerning the horizontal axis the higher the content of the methylammonium part in the perovskite structure. Also, with the increase in the content of the methylammonium part, the maxima and minima of Χ(h) decrease.

The obtained research results show that the perovskite composition has a significant impact on the surface topography of the thin film, while in each case, the entire tested surface is evenly covered with the perovskite material. Thanks to this, the obtained thin layers are uniform and free from breakthroughs to the substrate, which is extremely important from the point of view of further applications in optoelectronics.

### 3.2. Spectroscopy Characterization

#### 3.2.1. UV-Vis-NIR Spectroscopy

**Figure 7:**
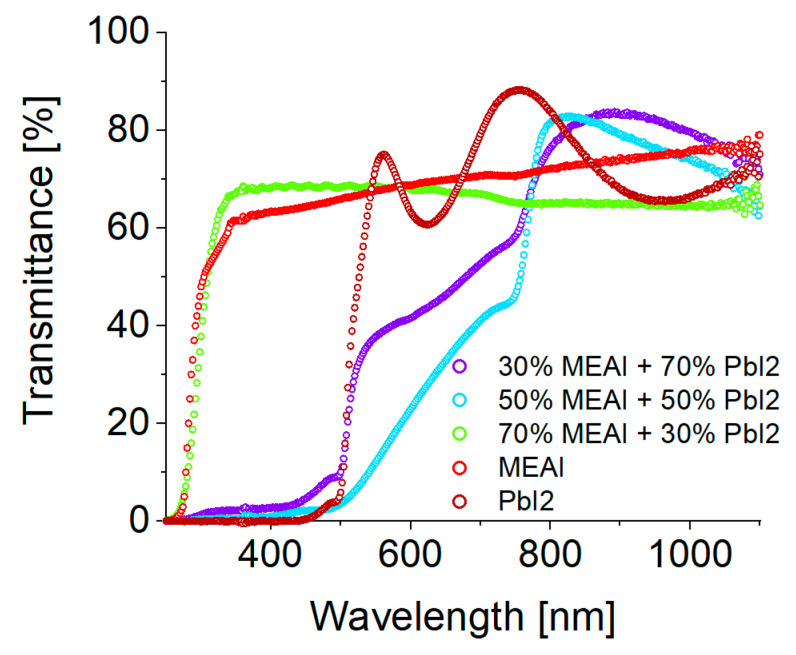
Graph showing experimental transmission spectra of MEAPbI_3_ perovskite thin films of three selected compositions and MEAI and PbI_2_ thin films made by PVco-D and PVD techniques, deposited on a glass substrate.

Figure 7 shows the optical transmission spectra of thin perovskite films of three different compositions and, additionally, the transmission spectra of thin MEAI and PbI_2_ films, which are included in the graph for comparison purposes. Perovskite structures were made using the PVco-D technique. MEAI and PbI_2_ structures were made using the PVD technique. All thin films were deposited on a transparent glass substrate. The obtained experimental transmission spectra of perovskite films with the composition of 30% MEAI + 70% PbI_2_ and 50% MEAI + 50% PbI_2_ can be divided into a region of strong absorption (range 250–740 nm) and weak absorption (range 740–1100 nm). This research result is consistent with the result obtained for perovskite crystals based on iodides provided by Green et al. [32]. Comparing both compositions, the second one is characterized by stronger absorption. The transmission spectrum of the perovskite with the composition of 70% MEAI + 30% PbI_2_ looks completely different. It is evenly flattened in the tested wavelength range, and the transmission level reaches almost 70% and slightly decreases with increasing wavelength.

**Figure 8:**
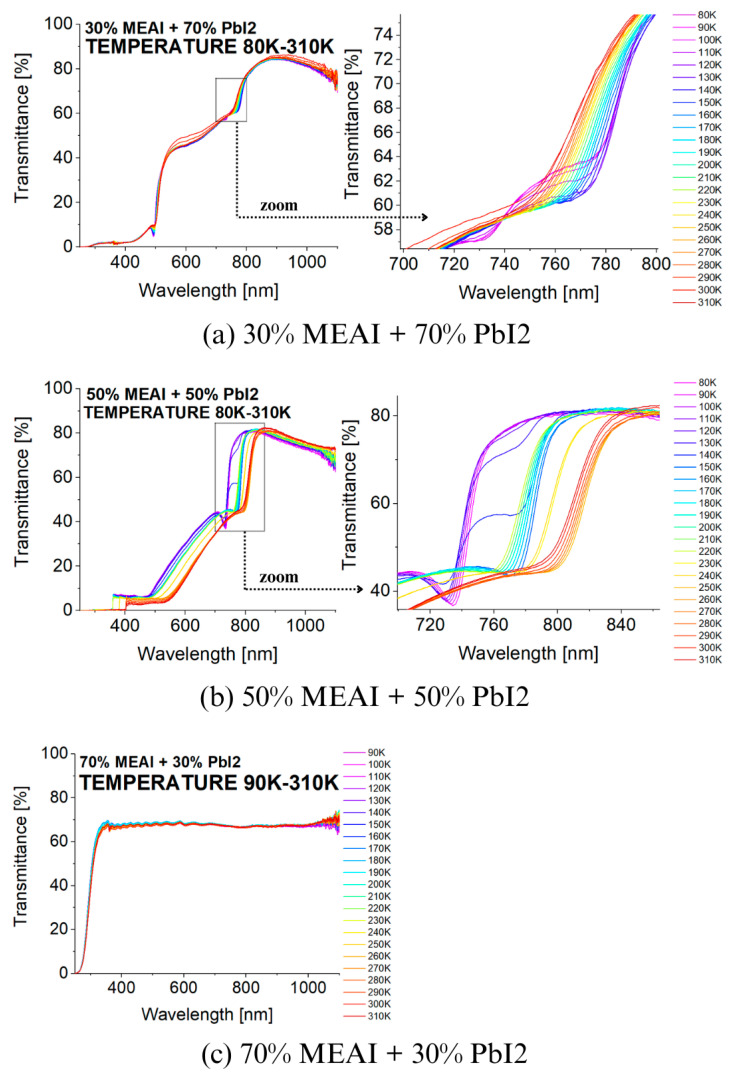
Temperature dependence of the transmission spectra of MEAPbI_3_ perovskite thin films of three different compositions produced by the PVco-D technique, deposited on a glass substrate: (**a**) 30% MEAI + 70% PbI_2_; (**b**) 50% MEAI + 50% PbI_2_; (**c**) 70% MEAI + 30% PbI_2_.

Figure 8 presents the transmission spectra of the tested perovskite materials recorded for different temperatures in the 80–310 K range. The spectra were recorded with a step of 10 K, which made it possible to observe changes caused by the influence of temperature. The changes in the transmission spectra induced by the temperature factor are closely related to the phase transitions occurring in the perovskite structures. The most interesting results were obtained for two of the three tested compositions of the MEAPbI_3_ perovskite, for the compositions of 30% MEAI + 70% PbI_2_ and 50% MEAI + 50% PbI_2_. For the third composition, no significant changes in the transmission spectra induced by the change in temperature were observed in the considered temperature and wavelength range.

Considering the effect of temperature on the first two compositions of the MEAPbI_3_ perovskite, it can be seen that changes in the course of the transmission spectra are visible in the entire studied wavelength range, while significant changes in the shape of the spectra are observed in the 700–850 nm range. Focusing in particular on this wavelength range, it can be seen that temperature changes cause shifts in the absorption band edges towards shorter or longer wavelengths. This happens alternately; sometimes, the changes are gentle, and other times, they occur abruptly. Such changes in the direction of shifts can be associated with phase transitions occurring in the studied materials (Table 2). In the case of the 30% MEAI + 70% PbI_2_ composition, two temperature ranges are observed: in the 80–140 K range, the spectrum shifts gently towards longer wavelengths, and in the 140–300 K range, a gentle shift in the spectrum towards shorter wavelengths is visible. At a temperature of 310 K, a sudden but not significant change towards longer wavelengths occurs. Based on these data, it is concluded that the phase transition from orthorhombic to tetragonal phase occurs at about 140 K, and the transition from tetragonal to cubic phase probably occurs at a temperature of about 310 K. The second perovskite composition for which significant changes are observed, 50% MEAI + 50% PbI_2_, shows slightly more changes in the transmission spectrum due to the action of temperatures from the wide range of 80 K to 310 K. Initially, in the temperature range of 80 K to 120 K, the transmission spectrum shifts to shorter wavelengths, then, in the range of 120 K to 150 K, towards longer wavelengths. Further, with increasing temperature up to 310 K, the spectrum tends to shift towards shorter wavelengths. Still, on the way, two sudden jumps to longer wavelengths are observed at temperatures around 220 K and 240 K (see Figure 8). On this basis, it is possible to determine the occurrence of the studied perovskite in the orthorhombic phase below the temperature of 120 K and the tetragonal phase above the temperature of 150 K. The temperature range from 120 K to 150 K is the range in which both phases can coexist, and, therefore, it is difficult to determine the exact single temperature value for which the phase change is observed.

Comparing the obtained data with the results described in the work of Green et al. [32] for the MAPbI3 perovskite in the form of a crystal, we see high agreement with the results for the sample with the composition of 50% MEAI + 50% PbI2. In the cited work, the most interesting changes in the spectroscopic spectrum occur in the same wavelength range, and the tendencies of the absorption spectrum shift are very similar. With increasing temperature, the spectrum shifts towards shorter wavelengths, except for the temperature range of 120 K–170 K, where a sizeable spectral jump towards longer wavelengths is observed. Green et al. define the phase transition temperature from the orthorhombic to the tetragonal phase of the crystalline MAPbI3 perovskite in the 120 K–170 K range. The range of these temperatures is defined as the coexistence of both phases. The research group of Kim et al. in another work assumed that the phase transitions of the MAPbI3 perovskite crystal occur at temperatures of 160 K for the transition from the orthorhombic to the tetragonal phase and at 330 K for the transition from the tetragonal to the cubic phase [33]. The differences regarding the temperatures at which phase transitions occur may primarily result from the fact that in this work, 2D low-dimensional structures are studied, while 3D crystalline structures are studied in the cited works.

#### 3.2.2. Photoluminescence Spectroscopy

**Figure 9:**
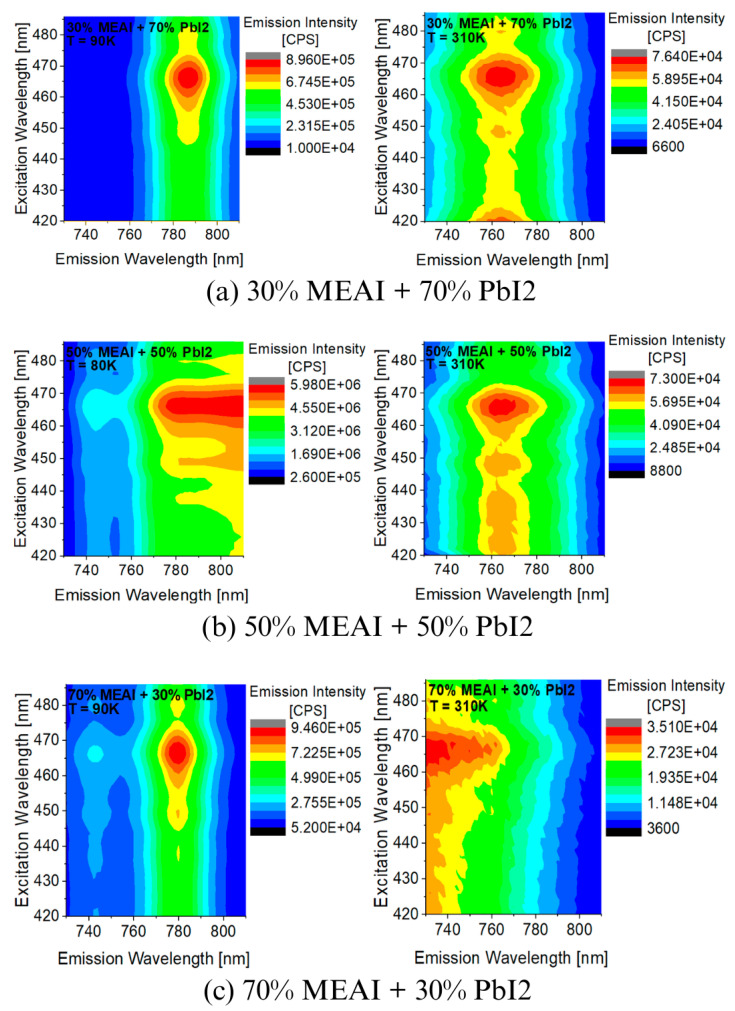
Experimental 3D photoluminescence spectra of MEAPbI_3_ perovskite thin films with different compositions recorded for temperatures of 80 K and 90 K and for a temperature of 310 K: (**a**) 30% MEAI + 70% PbI_2_; (**b**) 50% MEAI + 50% PbI_2_; (**c**) 70% MEAI + 30% PbI_2_. The excitation wavelength was λ_exc_ = 420 nm.

**Figure 10:**
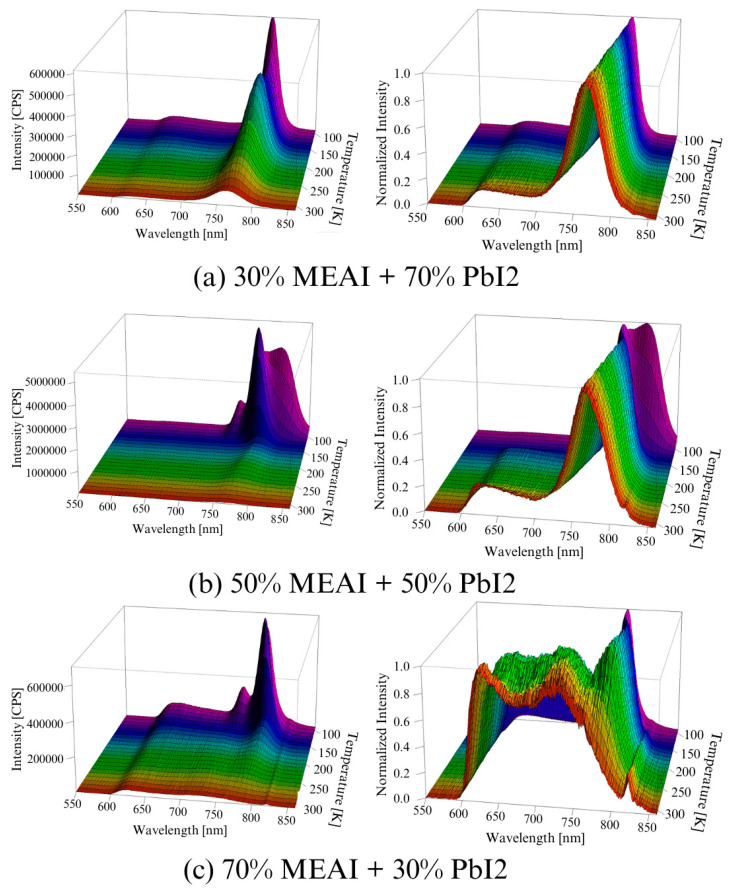
Experimental photoluminescence spectra of MEAPbI_3_ perovskite thin films with different compositions as a function of temperature: (**a**) 30% MEAI + 70% PbI_2_; (**b**) 50% MEAI + 50% PbI_2_; (**c**) 70% MEAI + 30% PbI_2_. The figure shows the spectra obtained directly from the measurement and the normalized spectra. The excitation wavelength was λ_exc_ = 420 nm.

Figure 9 and Figure 10 show experimental 3D PL spectra obtained for homogeneous thin films of MEAPbI_3_ perovskite with three different compositions. The studied materials were produced using the PVco-D technique on a transparent glass substrate. The excitation wavelength was λ_exc_ = 420 nm. Measurements were performed in the temperature range of 80–310 K for the 50% MEAI + 50% PbI_2_ composition and 90–310 K for the other two compositions. The measurements confirm that in the case of PL measurements, temperature also has a significant effect on the shape and intensity of the obtained spectra.

It can be seen directly from Figure 9 that the intensity of the PL spectra of each of the tested perovskite samples is higher at low temperatures; the positions of the excitation maxima are preserved regardless of the temperature, while the positions of the emission maxima change with the change in temperature. With the temperature increases from 90 K to 310 K, the maximum of the sample with the composition of 30% MEAI + 70% PbI_2_ shifts by about 20 nm towards shorter wavelengths. The situation is similar for the sample of 70% MEAI + 30% PbI_2_, whose emission maximum shifts by about 50 nm. For the third composition of 50% MEAI + 50% PbI_2_, the emission maximum changes position by about 30 nm towards shorter wavelengths when the temperature changes from 80 K to 310 K.

**Figure 11:**
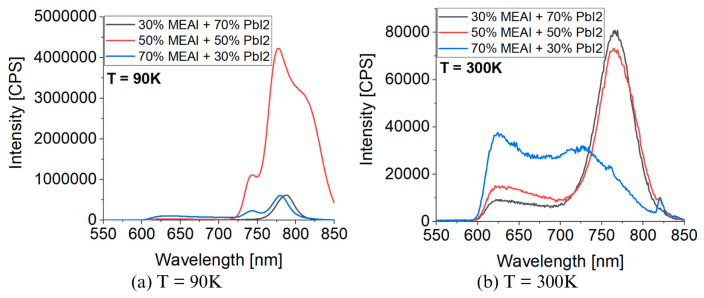
Comparison of photoluminescence spectra for different temperatures for MEAPbI_3_: (**a**) T = 90 K; (**b**) T = 300 K.

**Figure 12:**
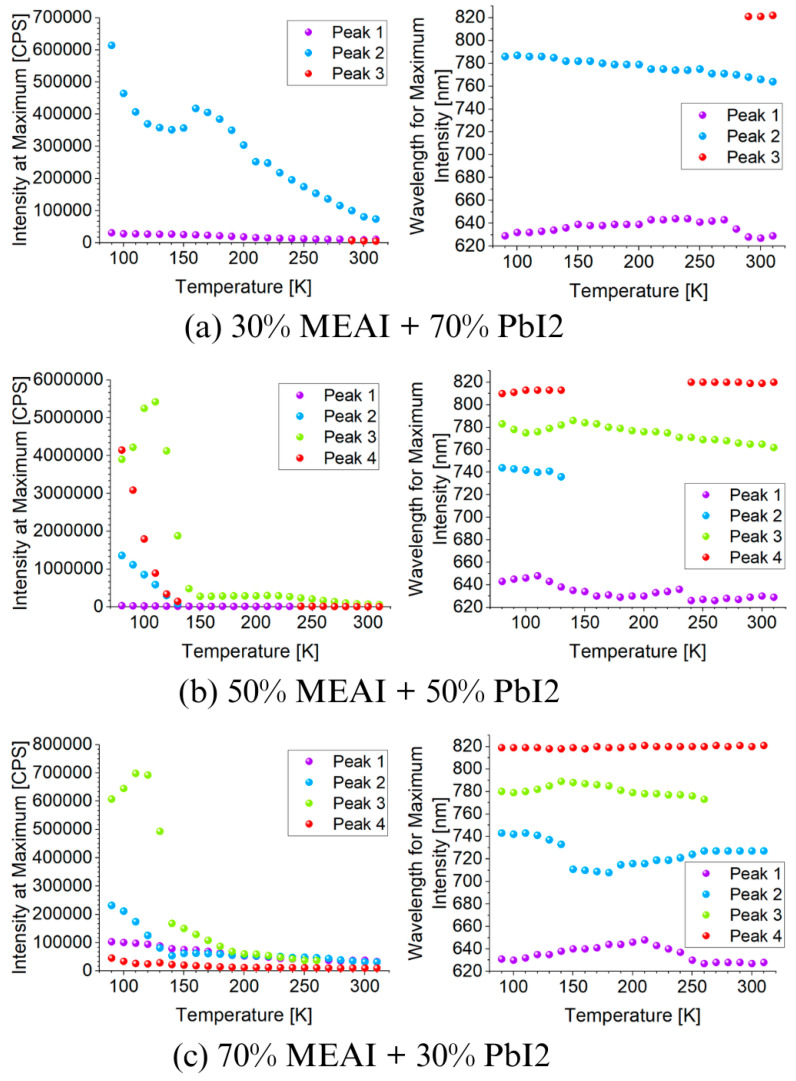
Dependence of the maximum intensity of peaks occurring in the photoluminescence spectra and the position of these peaks as a function of temperature for three different MEAPbI_3_ perovskite compositions: (**a**) 30% MEAI + 70% PbI_2_; (**b**) 50% MEAI + 50% PbI_2_; (**c**) 70% MEAI + 30% PbI_2_.

Figure 10 presents 3D graphs of the obtained PL spectra and the identical spectra but normalized. The differences in the presented images are visible at first glance. Each of the three tested samples reveals its specific features in the presented PL spectra. Common features are also observed, such as the maximum intensity of the PL spectrum in the low temperature region—for the compositions of 30% MEAI + 70% PbI_2_ and 50% MEAI + 50% PbI_2_, the maximum intensity is observed at 90 K, and for the composition of 70% MEAI + 30% PbI_2_, this is observed at 110 K. The peaks appearing in the spectra appear with a good approximation for the same wavelengths, except for the peak of ~743 nm, which is not observed in the sample with the composition of 30% MEAI + 70% PbI_2_. The number of observed peaks, intensity, and position change with the temperature changes. Similarly to changes induced by temperature change in UV-Vis-NIR transmission spectra, such changes can be associated with phase transitions occurring in the studied low-dimensional structures. In the case of the sample with 30% MEAI + 70% PbI_2_ composition in Figure 10a, three peaks are observed at ~629 nm, ~786 nm, and ~821 nm. Peak 2 is dominant here and, similarly to peak 1, is observed in the selected temperature range. Peak 3, of low intensity, appears only for higher temperatures (from 290 K). Four peaks are observed in the two remaining compositions at ~640 nm, ~743 nm, ~780 nm, and ~815 nm. For the 50% MEAI + 50% PbI_2_ sample, the dominant peaks are peaks 3 and 4, with peak 3 present in the entire selected temperature range and peak 4 observed for temperatures of 80 K–130 K and 240 K–310 K. For the 70% MEAI + 30% PbI_2_ sample, peaks 1, 2, and 3 are dominant: peaks 1 and 2 are visible in the entire temperature range, and peak 3 is in the 90 K–260 K range. For comparison purposes, Figure 11 shows the PL spectra of the tested samples, which were obtained for two temperatures—90 K and 300 K. An interesting observation is that at a temperature close to room temperature, the intensity of the peaks corresponding to the wavelength of ~780 nm is similar—especially for the samples with 30% MEAI + 70% PbI_2_ and 50% MEAI + 50% PbI_2_. However, at 90 K, the intensity of this peak determined for the sample with 50% MEAI + 50% PbI_2_ is about five times higher than for the other two samples. Based on the obtained PL spectra, graphs were made defining the dependence of the maximum intensity of peaks occurring in the PL spectra and the position of these peaks as a function of the temperature (Figure 12). From Figure 12, it is easy to see how many peaks are observed in the PL spectrum and at what temperatures. Considering this information and information on changes in the intensity of individual peaks, the phase transition temperatures can be determined for each tested sample (Table 2). The phase transition from the orthorhombic phase to the tetragonal phase in the sample with the composition of 30% MEAI + 70% PbI_2_ is observed in the temperature range of 140–160 K. The perovskite with this composition adopts the orthorhombic phase below 140 K and the tetragonal phase in the temperature range of 160 K–~RT (room temperature). At a temperature close to RT, another phase transition probably occurs from the tetragonal phase to the cubic phase, as evidenced by the appearance of peak 3 in the PL spectrum. The perovskite samples with the compositions of 50% MEAI + 50% PbI_2_ and 70% MEAI + 30% PbI_2_ also adopt the orthorhombic phase below 140 K, and above 140 K, they adopt the tetragonal phase. The temperature of 140 K is the phase-change temperature here.

Kong et al. obtained similar results based on PL spectra of CH_3_NH_3_PbI_3_ perovskite crystal [34]. Their work determined the transition temperature range from orthorhombic to tetragonal phase to be 120–150 K. In contrast, the studies presented in our work indicate that the temperature of this phase transition was determined based on PL spectra to be 140 K. Kong et al. also observed the prominent peaks in PL spectra around 750 nm, 780 nm, and 800 nm, with the dominant peak at 783 nm in RT. Their peaks are very similar to those described in this work. Studies of the linear thermal expansion of CH_3_NH_3_PbI_3_ perovskite crystals in a wide temperature range were also carried out by Keshavarz et al. [35]. The phase transition temperature that they determined was 160 K. The differences in the form of slightly different phase transition temperatures determined from the PL spectra from orthorhombic to tetragonal are probably the result of studying the material in different forms, i.e., in the form of a crystal and a thin-film structure.

## 4. Conclusions

In summary, in this paper, we described a novel approach to the traditional PVD process by using the co-deposition of more than one material simultaneously. We called this technique PVco-D. This approach allows for the obtention of low-dimensional perovskite structures with different chemical compositions distinguished by their homogeneity over larger surfaces. Based on the results of the research conducted, the following conclusions can be drawn:

1. The composition of the perovskite thin film has a significant impact on the structural and optical properties.
2. The increase in the MEAI content in the CH_3_NH_3_PbI_3_ perovskite structure is associated with more extensive and taller crystallites forming a thin layer and with higher roughness. The roughness of the perovskite layer increases by more than 60% with an increase in MEAI content from 30% to 70%.
3. The highest absorption of electromagnetic radiation in the wavelength range of 400–800 nm is demonstrated by the composition of 50% MEAI + 50% PbI_2_.
4. Measurements of UV-Vis-NIR spectra as a function of the temperature showed phase transitions occurring at temperatures of ~140 K and 310 K for the 30% MEAI + 70% PbI_2_ perovskite and in the temperature range of 120–150 K for the 50% MEAI + 50% PbI_2_ perovskite.
5. The PL spectrum measurements as a function of the temperature indicate phase transitions in the temperature range of 140 K–160 K and at RT for the perovskite with the composition of 30% MEAI + 70% PbI_2_, at 140 K for 50% MEAI + 50% PbI_2_ and 70% MEAI + 30% PbI_2_.

The temperature range used for spectroscopic measurements should be increased to determine the phase transition from tetragonal to cubic for the remaining compositions. According to theoretical assumptions, such a phase change occurs at temperatures close to RT or higher, even 350 K.

## Figures and Tables

**Table 1:** Average crystallite height and mean square roughness of MEAPbI_3_ perovskite thin films with different compositions and MEAI and PbI_2_ thin films.

Material	Average Crystallite Height [nm]	Mean Square Roughness [nm]
30% MEAI + 70% PbI_2_	18	4
50% MEAI + 50% PbI_2_	77	26
70% MEAI + 30% PbI_2_	109	20
MEAI	299	109
PbI_2_	34	16

**Table 2:** Phase transition temperatures determined from UV-Vis-NIR and PL spectroscopic measurements as a function of temperature.

Material	Phase Transition	Based on UV-Vis-NIR (T)	Based on PL (T)
30% MEAI + 70% PbI_2_	From orthorhombic to tetragonal	~140 K	140–160 K
	From tetragonal to cubic	310 K	~RT
50% MEAI + 50% PbI_2_	From orthorhombic to tetragonal	120–150 K	140 K
70% MEAI + 30% PbI_2_	From orthorhombic to tetragonal	Not found	140 K

## Data Availability

The data presented in this study are available upon request from the corresponding author. The data are not publicly available due to funding requirements.
